# Did Parents and Teachers Struggle with Child Survivors 20 Months after the 2011 Earthquake and Tsunami in Japan? A Retrospective Observational Study

**DOI:** 10.1371/journal.pone.0096459

**Published:** 2014-05-07

**Authors:** Masahide Usami, Yoshitaka Iwadare, Masaki Kodaira, Kyota Watanabe, Hirokage Ushijima, Tetsuya Tanaka, Maiko Harada, Hiromi Tanaka, Yoshinori Sasaki, Kazuhiko Saito

**Affiliations:** 1 Department of Child and Adolescent Psychiatry, National Center for Global Health and Medicine, Kohnodai Hospital, Ichikawa, Japan; 2 Department of Child Mental Health, Imperial Gift Foundation, Aiiku Hospital, Minato Japan; 3 Division of Neuropsychiaty, Department of Neuroscience, Yamaguchi University Graduate School of Medicine, Yamaguchi, Japan; 4 Institute of Women’s Health, Tokyo Women’s Medical University, Tokyo, Japan; 5 Department of Psychiatry, Tokyo Metropolitan Tama Medical Center, Tokyo, Japan; Old Dominion University, United States of America

## Abstract

**Background:**

On March 11, 2011, Japan was struck by the earthquake and tsunami. Twenty months after the disaster, we collected information on the difficulties faced by parents and teachers in dealing with the post-traumatic symptoms of child survivors. The aim of this study was to evaluate the relationship between post-traumatic symptoms in children and parents’ and teachers’ difficulties in dealing with children who survived the huge disaster.

**Methods:**

The subjects included 12,524 children from elementary, middle, and junior high schools in Ishinomaki City. The Post Traumatic Stress Symptoms for Children 15 items (PTSSC-15), a self-rating questionnaire on post-traumatic symptoms, was distributed among the children, and Strength and Difficulties Questionnaire (SDQ), a self-rating questionnaire on difficulties in dealing with children, was given to their parents and teachers. With PTSSC-15, a valid response was obtained from 10,909 (89.5%) participants. With SDQ for teachers and parents, valid responses were obtained from 10,577 (86.7%) and 7052 (83.9%) participants, respectively.

**Results:**

PTSSC-15 scores were significantly higher (P<0.001) in girls than in boys from the junior high school. These effect sizes were less than 0.30. Correlations of teachers’ SDQ scores and PTSSC-15 scores were significantly low (r<0.21, P<0.001) for both genders and all children. Likewise, correlations between SDQ scores assigned by parents (excluding “prosocial behavior”) and PTSSC-15 scores were significantly low (r<0.21, P<0.001) for both genders and at all school levels.

**Conclusion:**

This study elucidated that the difficulties faced by parents and teachers while dealing with child survivors significantly low correlate with the child’s post-traumatic symptoms caused by the 2011 earthquake and tsunami. Thus, it is important that clinicians should not only evaluate post-traumatic symptoms with a self-rating questionnaire but also try to objectively evaluate whether there were day-to-day difficulties caused by the post-traumatic symptoms.

## Introduction

On March 11, 2011, Japan was struck by a huge earthquake and tsunami. The tsunami caused tremendous damage and victimized many children [1], [2]. There have been many studies on the children who survived disasters [1]–[14]. After any disaster, post-traumatic stress disorder (PTSD) is the psychiatric diagnosis that should be considered most carefully by health care providers [3], [5], [7], [9]–[11], [15]–[20]. Post-traumatic symptoms tend to spontaneously heal over time; thus, the morbidity of PTSD is dependent on time, the subjects, and the diagnostic methods used [5], [7], [9], [10], [15]–[21]. The diagnostic criteria in the Diagnostic and Statistical Manual of Mental Disorders, 4^th^ edition, (DSM-IV) specify that patients experience significant difficulties in their daily lives because of their posttraumatic symptoms.

In a previous report, 8 months after the 2011 Japanese earthquake and tsunami, we collected information on the post-traumatic symptoms, sleep duration, and environmental damage conditions of children who lived through this tremendous disaster. That study demonstrated relationships of post-traumatic symptoms with gender, age, house damage, evacuation experience, and bereavement experience [1]. Furthermore, children with house damage and/or evacuation experiences exhibited significantly shorter sleep time than children without these experiences [2].

In the present work, 20 months after the earthquake and tsunami, we collected information on the difficulties faced by parents and teachers while dealing with the post-traumatic symptoms of child survivors. These data were gathered in the hope of a thorough investigation of the possible associations 20 months after exposure [5], [16], [21], [22].

Children with PTSD experience significant difficulties in their daily lives because of their post-traumatic symptoms. Therefore, we have not only evaluated the traumatic symptoms of child survivors, but also evaluated the difficulties in their daily lives. The aim of this study was to evaluate the relationship between post-traumatic symptoms in children and the difficulties faced by their parents and teachers in dealing with these children 20 months after the earthquake and tsunami. The main hypothesis was that child survivor’s traumatic symptoms caused day-to-day difficulties for parents and teachers. This hypothesis indicate child survivors with severe traumatic symptoms have severe day-to-day difficulties, and they may be diagnosed with PTSD.

We also evaluated the variations in the difficulties faced by parents and teachers. This is relevant because parents take care of children at home, and teachers interact with children at school: at different times of the day. The minor hypothesis was that the difficulties with children between parents and teachers were significantly different.

## Materials and Methods

### Study Design and Settings

This study involved observation of statistical associations of post-traumatic symptoms among children after the 2011 Japanese earthquake and tsunami. Ishinomaki City is the second largest city (population, 162,822) in Miyagi Prefecture, Japan. As of February 15, 2012, the death toll in Ishinomaki City was 3182, and 557 people were missing. The total number of collapsed houses and buildings, including half-collapsed houses, was 33,378, and 7298 temporary houses had been constructed.

### Recruitment and Participants

This survey was conducted as a part of the school education program conducted by the Board of Education of Ishinomaki City, Miyagi Prefecture. Survey sheets were distributed among all children who attended 43 elementary schools, and 21 junior high schools in Ishinomaki City. The survey was carried out in November 2012 (20 months after the 2011 disaster), after temporary housing had been provided for all evacuees in need in Ishinomaki City and after all evacuation centers had been closed.

First, the survey method was explained to the principals of all of the schools by the Education Committee of Ishinomaki City. Then teachers distributed a letter explaining the survey, which had been designed by the Education Committee, to all children and their parents. The letter clearly stated that if a student filled the questionnaire, it would be considered consent to the survey by both the parents and students. The letter also mentioned that the survey results would be used to provide children with psychological care to facilitate their education at school and that the results would be published as a scientific article. Informed consent was obtained when the students filled out the questionnaire.

This study was designed in accordance with the Declaration of Helsinki and approved by the Ethics Committee of the National Center for Global Health and Medicine. All participants gave written informed assent and their parents gave written informed consent after the procedure had been explained to them.

The Post Traumatic Stress Symptoms for Children 15 items (PTSSC-15), a self-rating questionnaire on post-traumatic symptoms, was distributed among 12,193 children registered at municipal schools in Ishinomaki City. The Questionnaire on Daily Life, a self-rating questionnaire that covers parameters such as time of waking and sleep onset, and eating/omission of breakfast.

A total of 12,193 copies of the Strength and Difficulties Questionnaire (SDQ) for teachers were distributed among the teachers of the same elementary, middle, and junior high school students in Ishinomaki City. The SDQ for parents was distributed to 8404 parents of elementary school (fourth to sixth grade) students and junior high school (seventh to ninth grade) students in Ishinomaki City.

The PTSSC-15 questionnaire and the Questionnaire on Daily Life were completed by 11,101 (91.0%) children. A valid response was obtained from 10,909 (89.5%) children.

Completed SDQs for teachers were obtained from 10,787 (88.4%) teachers. A valid response was obtained from 10,577 (86.7%) teachers.

Completed SDQs for parents were obtained from 7308 (87.0%) parents. A valid response was obtained from 7052 (83.9%) parents.

### Measures

A paper-based survey was conducted, asking questions regarding post-traumatic symptoms using a self-report form. The self-report form consisted of the PTSSC-15. The difficulties faced by parents and teachers were assessed using the SDQ score.

### PTSSC-15

PTSSC-15 is a self-rating questionnaire on stress reactions of children after a disaster. Post-traumatic Stress Symptoms 10 (PTSS10) [11], [22] had fewer questions and was used as a screening test after the Hanshin Great Earthquake; this instrument is widespread in Japan [1], [2], [11], [23]. In 105 Norwegian children (6–17 years old) devastated by the 2004 South East Asia Tsunami, PTSS10 was administered 10 and 30 months after the disaster [23], [24].

Each question is scored at 6 levels: 0 = completely disagree, 1 = mostly disagree, 2 = partially disagree, 3 = partially agree, 4 = mostly agree, and 5 = completely agree. Higher scores indicate more severe post-traumatic and depressive symptoms. Tomita et al. demonstrated the reliability and validity of PTSSC-15 in Japanese children and adolescents [24].

A previous study showed that PTSSC-15 scores are associated with the environmental damage caused by the 2011 Japanese tsunami [1].

### SDQ

The SDQ is a brief behavioral questionnaire for adults about 3- to 16-year-olds [25]. It exists in several versions: for researchers, clinicians, and educators. Each question is scored at 3 levels: 0 = not true, 1 = somewhat true, and 2 = certainly true.

SDQ tests 25 attributes, some of them positive and others negative. These 25 items are divided among 5 scales: emotional problems (5 items), conduct problems (5 items), hyperactivity/inattention (5 items), peer relationship problems (5 items), and prosocial behavior (5 items). The scores from the 4 problematic scales–emotional problems, conduct problems, hyperactivity/inattention, and peer relationship problems– are added up to produce a total difficulty score (based on 20 items).

Higher scores on emotional problems, conduct problems, hyperactivity/inattention, and peer relationship problems, and a higher total difficulty score indicate a more serious burden for parents or teachers. On the other hand, a higher score on prosocial behavior indicates better sociability. Matsuishi and coworkers demonstrated the reliability and validity of SDQ scores in Japanese children and adolescents [26].

### Statistical Analysis

#### Distribution of the PTSSC-15 and SDQ scores of parents and teachers

Kolmogorov-Smirnov tests were used to test the hypothesis that the distribution of the PTSSC-15 and SDQ scores of parents and teachers were normal. Parametric test was used when normal distribution was assumed and nonparametric test was used when the distribution differed significantly from normality.

#### PTSSC-15 score, school level, and gender

A previous study showed that the following factors influence the relationship between environmental damage conditions and the PTSC-15 scores: gender, age, house damage, evacuation experience, and bereavement experience. Therefore, children were divided into 3 grade groups: elementary school students (first to third grade), elementary school (fourth to sixth grade) students, and junior high school students (seventh to ninth grade). At each school level and in each gender, the median PTSSC-15 score and an interquartile range were determined.

#### SDQ scores of parents, school level, and gender

At 2 school levels (middle and junior high) and for both genders of the children, the parents’ SDQ median scores were determined (a child’s emotional problems, conduct problems, hyperactivity/inattention, peer relationship problems, the total difficulty score, and prosocial behavior).

#### SDQ scores of teachers, school level, and gender

At 2 school levels (middle and junior high) and for all children, the teachers’ SDQ median scores were determined (a child’s emotional problems, conduct problems, hyperactivity/inattention, peer relationship problems, the total difficulty score, and prosocial behavior).

#### Comparison between the parents’ and teachers’ SDQ scores

This was done separately for boys and girls by means of 2-way analysis of variance.

#### Correlations of the children’s PTSSC-15 scores with the SDQ scores of parents and teachers

Spearman’s correlation coefficients were calculated to assess if the self-rated problems (PTSSC-15 scores of the children) correlated with other-rated problems (the SDQ scores assigned by parents and teachers). In all tests, the significance level was 0.05 with 2-tailed analysis. All calculations were performed using PASW 18.0 and Prism 5 for Mac.

## Results

### PTSSC-15 Scores Based on School Level and Gender

Table 1 shows the PTSSC-15 scores after 20 months for each school level and gender. The PTSSC-15 score was significantly higher (P<0.001) in girls than in boys in junior high schools. These effect sizes were less than 0.30.

**Table 1:** Average PTSSC-15 scores of the children (by school level and gender).

	Gender											
	Male					Female						
	M	IR			N	M	IR			N	Effect size	P value
1st–3rd grade	15.0	5.0	–	26.0	1632	16.0	6.0	–	27.0	1581	0.03	ns
4th–6th grade	15.0	6.0	–	28.0	1792	17.0	7.0	–	28.0	1798	0.05	ns
7th–9th grade	18.0	8.0	–	29.0	1767	23.0	12.0	–	34.0	1789	0.29	<0.0001

Legend: M, median; IR, interquartile range; N, N:number of cases.

### SDQ Scores of Parents Based on School Level and Gender

The average SDQ scores of parents were compared among school levels and between genders of the children (Table 2). The “emotional symptoms” score in girls was significantly higher than that in boys [F(1, 6905) = 52.37, P<0.001]. The “conduct problems” score and “total difficulty score” in boys were significantly higher than those in girls [F(1, 7082) = 29.37, P<0.001 and F(1, 7082) = 18.69, P<0.001, respectively]. The “prosocial behavior” score in girls was significantly higher than that in boys [F(1, 7082) = 107.7, P<0.001]. Four subscores (excluding “peer relationship problems”) in elementary school (fourth to sixth grade) students were significantly higher than those in junior high school [F(1, 6905) = 26.72, P<0.001; F(1, 7082) = 66.09, P<0.001; F(1, 7082) = 60.97, P<0.001; F(1, 7082) = 44.65, P<0.001; and F(1, 7082) = 4.3097, P<0.05, respectively].

**Table 2:** Relationship between SDQ scores of parents based on school level and gender of children.

SDQ score of parent	Grade	Boys			Girls					
		M	SD	N	M	SD	N		F	P
Emotional problems	4th–6th	1.9	1.9	1777	2.1	2.2	1795	Gender×School level	9.618	**
	7th–9th	1.5	1.8	1655	2.0	2.1	1682	Gender	52.37	***
								School level	26.72	***
Conduct problems	4th–6th	2.2	1.7	1771	2.0	1.5	1795	Gender×School level	0.000	ns
	7th–9th	1.9	1.5	1838	1.7	1.5	1682	Gender	29.37	***
								School level	66.09	***
Hyperactivity/inattention	4th–6th	3.8	2.3	1771	3.8	2.1	1795	Gender×School level	0.0	ns
	7th–9th	3.4	2.2	1838	3.4	2.0	1682	Gender	0.0	ns
								School level	60.97	***
Peer relationship problems	4th–6th	2.0	1.8	1771	2.0	1.7	1795	Gender×School level	0.0	ns
	7th–9th	2.0	1.7	1838	2.0	1.7	1682	Gender	0.0	ns
								School level	0.0	ns
Total difficulty score	4th–6th	9.8	5.5	1771	9.0	5.5	1795	Gender×School level	3.862	*
	7th–9th	8.7	5.1	1838	8.4	5.3	1682	Gender	18.69	***
								School level	44.65	***
Prosocial behavior	4th–6th	5.9	2.0	1771	6.4	2.0	1795	Gender×School level	0.0	ns
	7th–9th	5.8	2.1	1838	6.3	2.0	1682	Gender	107.7	***
								School level	4.309	*

*p<0.05,

**p<0.001,

***p<0.0001.

### SDQ Scores of Teachers Based on School Level and Gender

The average SDQ scores of teachers were compared among school levels and between genders of the children (Table 3). “Conduct problems,” “hyperactivity/inattention,” “peer relationship problems,” and “total difficulty score” in boys were significantly higher than those in girls [F(1, 7337) = 218.9, P<0.001; F(1, 7337) = 760.4, P<0.001; F(1, 7337) = 13.84, P<0.001; and F(1, 7337) = 315.8, P<0.001, respectively]. The “prosocial behavior” score in girls was significantly higher than those in boys [F(1, 7337) = 271.3, P<0.001]. “Emotional problems” in junior high school students were significantly more pronounced than those in elementary school (fourth to sixth grade) students [F(1, 7337) = 13.45, P<0.001]. “Prosocial behavior” in elementary school (fourth to sixth grade) students was significantly better than that in junior high school students [F(1, 7337) = 24.42, P<0.001].

**Table 3:** Relationship between SDQ scores of teachers based on school level and gender.

SDQ score of teacher	Grade	Boys			Girls					
		M	SD	N	M	SD	N		F	P
Emotional problems	4th–6th	1.0	1.6	1865	1.0	1.7	1848	Gender×School level	1.494	ns
	7th–9th	1.1	1.8	1828	1.2	1.9	1800	Gender	1.494	ns
								School level	13.45	***
Conduct problems	4th–6th	1.5	1.8	1865	0.8	1.3	1848	Gender×School level	16.28	***
	7th–9th	1.4	1.8	1828	1.0	1.4	1800	Gender	218.9	***
								School level	1.809	ns
Hyperactivity/inattention	4th–6th	3.6	2.8	1865	1.8	1.9	1848	Gender×School level	19.78	***
	7th–9th	3.3	2.7	1828	2.0	2.1	1800	Gender	760.4	***
								School level	0.792	ns
Peer relationship problems	4th–6th	1.6	1.8	1865	1.4	1.6	1848	Gender×School level	1.538	ns
	7th–9th	1.6	1.8	1828	1.5	1.7	1800	Gender	13.84	***
								School level	1.538	ns
Total difficulty score	4th–6th	7.7	5.9	1865	5.0	4.9	1848	Gender×School level	9.551	**
	7th–9th	7.4	6.0	1828	5.5	5.3	1800	Gender	315.8	***
								School level	0.597	ns
Prosocial behavior	4th–6th	5.2	2.6	1865	6.3	2.5	1848	Gender×School level	2.713	ns
	7th–9th	5.0	2.7	1828	5.9	2.6	1800	Gender	271.3	***
								School level	24.42	***

*p<0.05,

**p<0.001,

***p<0.0001.

### SDQ Scores of Parents and Teachers Based on the Child’s Gender

The average SDQ scores assigned by parents and teachers were compared by rater and based on the child’s gender (Table 4). In elementary school (fourth to sixth grade) students, “emotional problems,” “conduct problems,” “hyperactivity/inattention,” and “total difficulty score” in boys were significantly higher than those in girls [F(1, 7281) = 5.259, P<0.05; F(1, 7281) = 146.3, P<0.001; F(1, 7281) = 278.0, P<0.001; F(1, 7281) = 6.102, P<0.05; and F(1, 7281) = 186.7, P<0.001, respectively]. “Prosocial behavior” in girls was better than that in boys [F(1, 7281) = 220.5, P<0.001].

**Table 4:** Differences between SDQ scores by raters and by gender.

SDQ			Gender								
			Boys			Girls					
			M	SD	N	M	SD	N		F	P
4th–6th	Emotional problems	Parent	1.9	1.9	1777	2.1	2.2	1795	Gender×Rater	5.259	*
		Teacher	1.0	1.6	1865	1.0	1.7	1848	Gender	5.259	*
									Rater	525.9	***
	Conduct problems	Parent	2.2	1.7	1771	2.0	1.5	1795	Gender×Rater	45.16	***
		Teacher	1.5	1.8	1865	0.8	1.3	1848	Gender	146.3	***
									Rater	652.2	***
	Hyperactivity/inattention	Parent	3.8	2.3	1771	3.8	2.1	1795	Gender×Rater	278.0	***
		Teacher	3.6	2.8	1865	1.8	1.9	1848	Gender	278.0	***
									Rater	415.3	***
	Peer relationship problems	Parent	2.0	1.8	1771	2.0	1.7	1795	Gender×Rater	6.102	*
		Teacher	1.6	1.8	1865	1.4	1.6	1848	Gender	6.102	*
									Rater	152.5	***
	Total difficulty score	Parent	9.8	5.5	1771	9.0	5.5	1795	Gender×Rater	55.02	***
		Teacher	7.7	5.9	1865	5.0	4.9	1848	Gender	186.7	***
									Rater	567.2	***
	Prosocial behavior	Parent	5.9	2.0	1771	6.4	2.0	1795	Gender×Rater	31.01	***
		Teacher	5.2	2.6	1865	6.3	2.5	1848	Gender	220.5	***
									Rater	55.14	***
7th–9th	Emotional problems	Parent	1.5	1.8	1655	2.0	2.1	1682	Gender×Rater	19.22	***
		Teacher	1.1	1.8	1828	1.2	1.9	1800	Gender	43.23	***
									Rater	172.9	***
	Conduct problems	Parent	1.9	1.5	1838	1.7	1.5	1682	Gender×Rater	7.344	**
		Teacher	1.4	1.8	1828	1.0	1.4	1800	Gender	66.10	***
									Rater	264.4	***
	Hyperactivity/inattention	Parent	3.4	2.2	1838	3.4	2.0	1682	Gender×Rater	146.1	***
		Teacher	3.3	2.7	1828	2.0	2.1	1800	Gender	146.1	***
									Rater	194.5	***
	Peer relationship problems	Parent	2.0	1.7	1838	2.0	1.7	1682	Gender×Rater	1.498	ns
		Teacher	1.6	1.8	1828	1.5	1.7	1800	Gender	1.498	ns
									Rater	121.3	***
	Total difficulty score	Parent	8.7	5.1	1838	8.4	5.3	1682	Gender×Rater	38.62	***
		Teacher	7.4	6.0	1828	5.5	5.3	1800	Gender	73.01	***
									Rater	266.1	***
	Prosocial behavior	Parent	5.8	2.1	1838	6.3	2.0	1682	Gender×Rater	12.65	***
		Teacher	5.0	2.7	1828	5.9	2.6	1800	Gender	155.0	***
									Rater	113.9	***

*p<0.05,

**p<0.001,

***p<0.0001.

In junior high school students, “emotional problems,” “conduct problems,” “hyperactivity/inattention,” and “total difficulty score” in boys were significantly higher than those in girls [F(1, 7281) = 43.23, P<0.001; F(1, 7281) = 66.10, P<0.001; F(1, 7281) = 146.1, P<0.001; and F(1, 7281) = 73.01, P<0.001, respectively]. “Prosocial behavior” score in girls was significantly higher than that in boys [F(1, 7281) = 155.0, P<0.001].

At all school levels, “emotional problems,” “conduct problems,” “hyperactivity/inattention,” and “total difficulty score” assigned by parents were significantly higher than those assigned by teachers [all F(1, 7281) >55.14, P for all<0.001].

### Correlations between PTSSC-15 Scores and SDQ Scores Assigned by Parents and Teachers

Correlations between SDQ scores assigned by parents and teachers and the PTSSC-15 scores are shown in Table 5. The SDQ scores assigned by teachers were significantly low correlated with the PTSSC-15 scores in both genders and at all school levels (all r <0.21, P for all<0.001). Similarly, the SDQ scores assigned by parents (excluding “prosocial behavior”) were significantly low correlated with the PTSSC-15 scores in both genders and at all school levels (all r<0.31, P for all<0.001). The “prosocial behavior” scores assigned by parents were not correlated with the PTSSC-15 scores in both gender and at all school levels (r = −0.00, r = −0.02, r = −0.02, and r = −0.00, respectively).

**Table 5:** Relationships between SDQ scores of teachers and children’s PTSSC-15 scores.

SDQ	Grade	PTSSC-15 of parent				PTSSC-15 of teacher			
		Boys		Girls		Boys		Girls	
Emotional problems	1th–3th					0.13	***	0.13	***
	4th–6th	0.31	***	0.27	***	0.17	***	0.17	***
	7th–9th	0.25	***	0.31	***	0.21	***	0.21	***
Conduct problems	1th–3th					0.12	***	0.12	***
	4th–6th	0.22	***	0.21	***	0.09	***	0.09	***
	7th–9th	0.15	***	0.19	***	0.05	***	0.05	***
Hyperactivity/inattention	1th–3th					0.15	***	0.15	***
	4th–6th	0.22	***	0.21	***	0.15	***	0.15	***
	7th–9th	0.15	***	0.19	***	0.11	***	0.11	***
Peer relationship problems	1th–3th					0.13	***	0.13	***
	4th–6th	0.23	***	0.17	***	0.13	***	0.13	***
	7th–9th	0.17	***	0.17	***	0.14	***	0.14	***
Total difficulty score	1th–3th					0.18	***	0.18	***
	4th–6th	0.34	***	0.29	***	0.19	***	0.19	***
	7th–9th	0.24	***	0.30	***	0.16	***	0.16	***
Prosocial behavior	1th–3th					−0.08	*	−0.08	*
	4th–6th	0.00		−0.02		−0.04	*	−0.04	*
	7th–9th	−0.02		−0.00		−0.06	*	−0.06	*

*p<0.05,

**p<0.001,

***p<0.0001.

## Discussion

This study explored the associations between post-traumatic symptoms of children and the difficulties faced by their parents and teachers 20 months after the 2011 earthquake and tsunami. This study elucidated that the burden faced by parents and teachers while handling child survivors significantly low correlate with the child’s post-traumatic symptoms. The main hypothesis (that the above associations exist) was rejected after a careful analysis of the data. Our results imply that after a major disaster, relying on a self-rating questionnaire as the only screening tool for PTSD may result in an inflated number of children who appear to be at high risk of PTSD. However, most children who appear to be at a high risk of PTSD may not experience problems in their daily activities, according to our findings. The diagnostic criteria of PTSD in DSM-IV include substantial difficulties in day-to-day activities as a result of the post-traumatic symptoms. Thus, despite the large number of children who experience post-traumatic symptoms and stressful experiences after a big natural disaster, only a few of them may qualify for a diagnosis of PTSD.

The minor hypothesis that the difficulties with children between parents and teachers were significantly different was confirmed. The difficulties faced by parents were significantly more than those faced by teachers. This result may be explained by the fact that parents take care of children at home whereas teachers interact with children only at school: different times of the day and different duration of the interactions. We also uncovered substantial differences in post-traumatic symptoms and in adult’s difficulties that are related to the gender and age of the children. The adult’s difficulties faced by parents and teachers while handling child survivors did not correlate with post-traumatic symptoms of these children. Therefore, these children would not have diagnosis of PTSD. Our findings are expected to improve the diagnosis of PTSD in pediatric population, especially those affected by natural disasters.

## Limitations

This study was a survey with a self-rating questionnaire carried out in only 1 district in Japan and it is therefore impossible to calculate the morbidity of PTSD in children after the 2011 Japanese earthquake and tsunami based on the results of this survey. Therefore, this study is insufficient as an epidemiological survey for psychiatric diagnosis. Examinations by child psychiatrists using operational diagnostic criteria and structured interviews are necessary for accurate psychiatric diagnosis. In addition, the results of this study on children in Ishinomaki City do not reflect all characteristics of children who experienced the 2011 Japanese earthquake and tsunami.

## Conclusion

This study elucidated that the burden faced by parents and teachers while handling child survivors did significantly low correlate with post-traumatic symptoms of these children. The difficulties experienced while handling these children were significantly more for the parents than for the teachers. This indicates that clinicians should not only evaluate post-traumatic symptoms with a self-rating questionnaire but also try to objectively evaluate whether there were day-to-day difficulties caused by the post-traumatic symptoms.

## References

[1] Usami M, Usami M, Iwadare Y, Iwadare Y, Kodaira M, et al (2012) Relationships between Traumatic Symptoms and Environmental Damage Conditions among Children 8 Months after the 2011 Japan Earthquake and Tsunami. PLoS ONE 7: e50721 10.1371/journal.pone.0050721.t007 23209817 PMC3510162

[2] Usami M, Iwadare Y, Kodaira M, Watanabe K, Aoki M, et al (2013) Sleep Duration among Children 8 Months after the 2011 Japan Earthquake and Tsunami. PLoS ONE 8: e65398 10.1371/journal.pone.0065398.t008 23738015 PMC3667796

[3] Chemtob CM, Nomura Y, Abramovitz RA (2008) Impact of conjoined exposure to the World Trade Center attacks and to other traumatic events on the behavioral problems of preschool children. Arch Pediatr Adolesc Med 162: 126–133 10.1001/archpediatrics.2007.36 18250236

[4] Piyasil V, Ketuman P, Plubrukarn R, Jotipanut V, Tanprasert S, et al (2007) Post traumatic stress disorder in children after tsunami disaster in Thailand: 2 years follow-up. J Med Assoc Thai 90: 2370–2376. 18181322

[5] Ma X, Liu X, Hu X, Qiu C, Wang Y, et al (2011) Risk indicators for post-traumatic stress disorder in adolescents exposed to the 5.12 Wenchuan earthquake in China. Psychiatry Res 189: 385–391 10.1016/j.psychres.2010.12.016 21295350

[6] Piyasil V, Ketumarn P, Ularntinon S (2008) Post-traumatic stress disorder in Thai children living in area affected by the tsunami disaster: a 3 years followup study. ASEAN Journal of Psychiatry 8: 99–103.

[7] WHO.Mental Health Assistance to the Populations Affected by the Tsunami in Asia. Available: http://www.who.int/mental_health/resourc es/tsunami/en/. Accessed: 3 Jun 2010.

[8] Ularntinon S, Piyasil V, Ketumarn P, Sitdhiraksa N, Pityaratstian N, et al (2008) Assessment of psychopathological consequences in children at 3 years after tsunami disaster. J Med Assoc Thai 91 Suppl 3 S69–S75. 19253499

[9] WHO. Tsunami Wreaks Mental Health Havoc. 1 June 2005. Available: http://www.who.int/bulletin/volumes/83/6/infocus0605/en/index.html. Accessed: 29 Oct 2012.

[10] Hafstad GS, Kilmer RP, Gil-Rivas V (2011) Posttraumatic growth among Norwegian children and adolescents exposed to the 2004 tsunami. Psychological Trauma: Theory, Research, Practice, and Policy 3: 130–138 10.1037/a0023236

[11] Kato H, Asukai N, Miyake Y (1996) Post-traumatic symptoms among younger and elderly evacuees in the early stages following the 1995 Hanshin Awaji earthquake in Japan. Acta Psychiatrica 93(6), 477–481.doi:10.1111/j.1600-0447.1996.tb10680.x. 10.1111/j.1600-0447.1996.tb10680.x 8831865

[12] Becker SM (2007) Psychosocial Care for Adult and Child Survivors of the Tsunami Disaster in India. J Child Adolescent Psych Nursing 20: 148–155 10.1111/j.1744-6171.2007.00105.x 17688552

[13] Manuel Carballo BH, Hernandez M (2005) Psychosocial aspects of the Tsunami. Journal of the Royal Society of Medicine 98: 396. 16140849 10.1258/jrsm.98.9.396 PMC1199633

[14] Du YB, Lee CT, Christinam D, Belfer ML, Betancourt TS, et al (2011) The living environment and children’s fears following the Indonesian tsunami. Disasters 36: 495–513 10.1111/j.1467-7717.2011.01271.x 22098206 PMC3991231

[15] Mullett-Hume E, Anshel D, Guevara V, Cloitre M (2008) Cumulative trauma and posttraumatic stress disorder among children exposed to the 9/11 World Trade Center attack. Am J Orthopsychiatry 78: 103–108 10.1037/0002-9432.78.1.103 18444732

[16] Jia Z, Tian W, He X, Liu W, Jin C, et al (2010) Mental health and quality of life survey among child survivors of the 2008 Sichuan earthquake. Qual Life Res 19: 1381–1391 10.1007/s11136-010-9703-8 20623339

[17] Weisz JR, Jensen AL (2001) Child and adolescent psychotherapy in research and practice contexts: Review of the evidence and suggestions for improving the field. Eur Child Adolesc Psychiatry 10: S12–S18 10.1007/s007870170003 11794552

[18] Dyb G, Jensen TK, Nygaard E (2011) Children’ and parents’ posttraumatic stress reactions after the 2004 tsunami. Clinical Child Psychology and Psychiatry 16: 621–634 10.1177/1359104510391048 21565871

[19] Kim B, Kim J, Kim H, Shin M, Cho S, et al (2009) A 6-month follow-up study of posttraumatic stress and anxiety/depressive symptoms in korean children after direct or indirect exposure to a single incident of trauma. J Clin Psychiatry 70: 1148–1154 10.4088/JCP.08m04896 19538906

[20] Wiguna T, Guerrero APS, Kaligis F, Khamelia M (2010) Psychiatric morbidity among children in North Aceh district (Indonesia) exposed to the 26 December 2004 tsunami. Asia-Pacific Psychiatry 2: 151–155 10.1111/j.1758-5872.2010.00079.x

[21] Trickey D, Siddaway AP, Meiser-Stedman R, Serpell L, Field AP (2012) A meta-analysis of risk factors for post-traumatic stress disorder in children and adolescents. Clinical Psychology Review 32: 122–138 10.1016/j.cpr.2011.12.001 22245560

[22] Weisaeth L (1989) Torture of a Norwegian ship’s crew. The torture, stress reactions and psychiatric after-effects. Acta Psychiatr Scand Suppl 355: 63–72. 2624136

[23] Jensen TK, Dyb G, Nygaard E (2009) A Longitudinal Study of Posttraumatic Stress Reactions in Norwegian Children and Adolescents Exposed to the 2004 Tsunami. Arch Pediatr Adolesc Med 163: 856 10.1001/archpediatrics.2009.151 19736341

[24] Yoshiki T, Tetsu T, Ryuzou Y, Katsuhiko S, Nobuo K, et al (2002) The reliability and validity of Post Traumatic Stress symptoms for Children-15 items(PTSSC-15). The Journal of Human Development and Clinical Psychology 8: 29–36.

[25] sdqinfo.com (n.d.) sdqinfo.com. Available: http://www.sdqinfo.com. Accessed: 30 Jun 2013.

[26] Matsuishi T, Nagano M, Araki Y, Tanaka Y, Iwasaki M, et al (2008) Scale properties of the Japanese version of the Strengths and Difficulties Questionnaire (SDQ): A study of infant and school children in community samples. Brain Dev 30: 410–415 10.1016/j.braindev.2007.12.003 18226867

